# Radiation produces differential changes in cytokine profiles in radiation lung fibrosis sensitive and resistant mice

**DOI:** 10.1186/1756-8722-2-6

**Published:** 2009-02-02

**Authors:** Xiaoping Ao, Lujun Zhao, Mary A Davis, David M Lubman, Theodore S Lawrence, Feng-Ming Kong

**Affiliations:** 1 Department of Radiation Oncology, University of Michigan, Ann Arbor, MI 48109, USA; 2 Department of Surgery, University of Michigan, Ann Arbor, MI 48109, USA

## Abstract

**Background:**

Recent research has supported that a variety of cytokines play important roles during radiation-induced lung toxicity. The present study is designed to investigate the differences in early cytokine induction after radiation in sensitive (C57BL/6) and resistant mice (C3H).

**Results:**

Twenty-two cytokines in the lung tissue homogenates, bronchial lavage (BAL) fluids, and serum from 3, 6, 12, 24 hrs to 1 week after 12 Gy whole lung irradiation were profiled using a microsphere-based multiplexed cytokine assay. The majority of cytokines had similar baseline levels in C57BL/6 and C3H mice, but differed significantly after radiation. Many, including granulocyte colony-stimulating factor (G-CSF), interleukin-6 (IL-6), and keratinocyte-derived chemokine (KC) were elevated significantly in specimens from both strains. They usually peaked at about 3–6 hrs in C57BL/6 and 6–12 hrs in C3H. At 6 hrs in lung tissue, G-CSF, IL-6, and KC increased 6, 8, and 11 fold in C57BL/6 mice, 4, 3, and 3 fold in the C3H mice, respectively. IL-6 was 10-fold higher at 6 hrs in the C57BL/6 BAL fluid than the C3H BAL fluid. MCP-1, IP-10, and IL-1α also showed some differences between strains in the lung tissue and/or serum. For the same cytokine and within the same strain of mice, there were significant linear correlations between lung tissue and BAL fluid levels (R^2 ^ranged 0.46–0.99) and between serum and tissue (R^2 ^ranged 0.56–0.98).

**Conclusion:**

Radiation induced earlier and greater temporal changes in multiple cytokines in the pulmonary fibrosis sensitive mice. Positive correlation between serum and tissue levels suggests that blood may be used as a surrogate marker for tissue.

## Background

Radiation-induced pulmonary injury to normal lung tissue is a dose-limiting complication for cancer patients receiving radiotherapy to the chest region [1-3]. Depending on both radiation dose and volume, lung injury is characterized by inflammation associated pneumonitis which may progress to permanent pulmonary fibrosis. An improved understanding of the factors leading to pneumonitis and fibrosis could result in an increased ability to predict which patients are likely to develop the disease so that they could receive appropriate treatment.

The response to ionizing radiation involves a number of mediators including inflammatory cytokines produced by macrophages, epithelial cells, and fibroblasts [4,5]. An early activation of an inflammatory reaction can lead to the expression and maintenance of a perpetual cytokine cascade, resulting in increased collagen production and ultimately fibrosis [6]. For example the cytokine, transforming growth factor-beta1 (TGF-β1), is thought to be a key mediator of lung toxicity and may predict resultant damage to normal lung following radiation [7,8]. Since a complex cytokine network initiates and sustains the inflammatory and fibrogenic processes associated with radiation-induced lung injury [9], the ability to simultaneously quantify multiple cytokines is critical for deciphering how they affect radiation-induced lung toxicity. One such assay, a microsphere-based sandwich immunoassay for flow cytometry, is a highly sensitive and selective multiplexed assay platform to simultaneously measure many cytokines in low volume samples, e.g. 25 μL sample for 22 mouse cytokines/chemokines [10]. This assay platform, the most comprehensive one available on the market during the time of our experiment, provides a powerful tool for multiple cytokine profiling and a more complete picture of the complex cytokine network.

The present study was designed to take advantage of this platform and the known differences between the C57BL/6 and C3H mouse strains in their response to lung radiation [11-14]. C57BL/6 mice are much more sensitive to radiation-induced pulmonary fibrosis than C3H mice [15]. Johnston et al. have extensively studied the mRNA expression of different cytokines in mouse lung after ionizing radiation [6,16-18]; these studies focused on the remodeling phase but not the initial response. Others noted that cytokine mRNA elevation occurred early after radiation [19,20], and an early study on TGF-β1 showed a rapid induction of immunoreactivity in tissue at 1 hour post radiation [21]. While most of the previous multiplexed cytokine studies focused on the transcriptional mRNAs instead of cytokine proteins, proteins, rather than mRNA, are the actual biological effectors, making it likely that cytokine levels will better correlate with biological outcome than mRNA levels. Therefore, we focused our study on the cytokines themselves. We hypothesized that there would be significant differences in cytokine profiles immediately after radiation in these two strains of mice with different sensitivities to radiation. We also hypothesized that serum cytokine profiles would correlate with lung tissue levels such that a panel of serum markers could be developed which predict for radiation-induced lung toxicity. Therefore, in this study, we treated C57BL/6 and C3H mice with thoracic radiation and, utilizing the multiplex immunoassay platform, measured the levels of 22 cytokines in lung tissue, broncheoalveolar lavage fluid (BAL), and serum at times from 3 hrs to 1 week after radiation.

## Methods and materials

### Animals and radiation treatment

Five to 6 week-old male C57BL/6 and C3H mice were purchased from Charles River Breeding Labs (Wilmington, MA). A plastic jig was used to restrain the mice without anesthesia, and lead strips were placed to shield the head and abdomen. A Phillips 250 orthovoltage unit was used to deliver 12 Gy at 143.27 cGy/min to the thorax. The field size (2 × 3 cm) was set to provide adequate coverage of the whole lung. Dosimetry was carried out using an ionization chamber connected to an electrometer system, which is directly traceable to a National Institute of Standards and Technology calibration. The use of animals was in compliance with the regulations of the University of Michigan and with NIH guidelines. The susceptibility of the C57BL/6 mouse strain to radiation-induced lung damage [11] has been confirmed in our laboratory by measurement of lung function via plethysmography at 8 weeks post radiation [22].

### Specimen preparation

Lung tissue, bronchial lavage (BAL) fluid, and blood samples were collected from controls and at 3 hrs, 6 hrs, 12 hrs, 24 hrs, and 1 week after radiation (3 mice at each time point for each strain). Blood was drawn from anesthetized mice via cardiac puncture followed by portal venous perfusion with 20 ml PBS. The right lung was lavaged with 500 μL saline, BAL fluid was then obtained (about 300 μL each animal). The left lung which was used for cytokine measurement was quickly frozen in 70% ethanol containing dry ice. Blood was allowed to sit for 4 hrs at room temperature to allow clotting, and the supernatant (serum) was collected after centrifugation. Serum was used as we were also interested in assessing level of TNF-α in this study. All samples were stored at -80°C until assay. At the time of analysis, 25–40 mg of frozen lung tissue was aliquoted using an Ohaus analytical balance, which can measure weight accurately to 0.1 mg. The frozen tissue was then disrupted and homogenized in 200 μL tissue lysis buffer (CelLytic™ MT Mammalian Tissue Lysis/Extraction Reagent from Sigma-Aldrich) using a tissue grinder (Duall^® ^All-Glass from Kimble/Kontes). After homogenization, the samples were centrifuged at 10,000 × g for 5 min, and the supernatants were used for cytokine profiling.

### Multiplexed cytokine analysis

The cytokine concentrations in the serum, BAL fluids, and lung tissue lysates were assayed using a Mouse Cytokine/Chemokine Lincoplex kit (Linco Research, St. Charles, Missouri). The kit can simultaneously quantify 22 mouse cytokines and chemokines: Interleukin (IL)-1α, IL-1β, IL-2, IL-4, IL-5, IL-6, IL-7, IL-9, IL-10, IL-12p70, IL-13, IL-15, IL-17, Interferon-γ (IFN-γ), Interferon γ-inducible Protein-10 (IP-10), Granulocyte Colony-Stimulating factor (G-CSF), Granulocyte Macrophage Colony-Stimulating Factor (GM-CSF), Tumor Necrosis Factor-α (TNF-α), keratinocyte-derived chemokine (KC), Monocyte Chemoattractant Protein-1 (MCP-1), Macrophage Inflammatory Protein-1α(MIP-1α), and Regulated upon Activation, Normal T-cell Expressed, and Secreted (RANTES). The kit contains spectrally distinct antibody-immobilized beads (22 bead sets specifically for the above cytokines), cytokine standard cocktail, cytokine quality control I and II, detection antibody cocktail, streptavidin-phycoerythrin, assay buffer, wash buffer, serum matrix, and a microtiter filter plate.

The assay was performed according to the manufacturer's protocol. Tissue lysis buffer, saline, and serum matrix were used as the sample matrices for tissue lysates, BAL fluids, and serum, respectively. After preparation, samples were processed (50 beads per bead set in 50 μL sample size) on a Luminex 100 instrument (Luminex Corporation, Austin, TX). All the samples were run in duplicate. The detection limit of this kit is 3.2 pg/ml for all the included cytokines.

### Statistical analysis

Data are presented as mean ± standard error of the mean (SEM). One way ANOVA from Origin 7.0 was used to compare the significance between two sets of data. Values were considered significantly different when *p *< 0.05.

## Results

### Cytokine levels in lung tissue lysates

**Figure 1:**
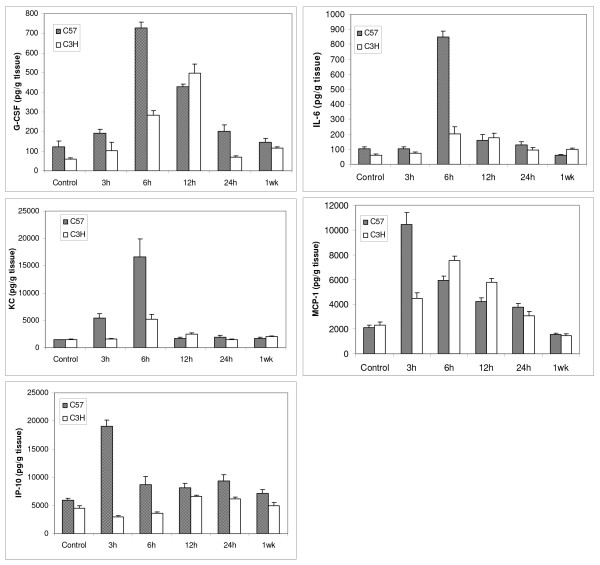
**Mouse lung tissue cytokine levels in C57BL/6 (C57) and C3H mice**. Mice were untreated or received a single dose of 12 Gy to the lung. Cytokine levels were normalized based on lung tissue mass. Data are expressed as the mean ± SEM of duplicate determinations from three different mice for each time point of each strain.

We began by analyzing cytokine levels in the lungs of control mice. Nine cytokines out of the 22 measured in the lung (GM-CSF, G-CSF, IL-6, IL-9, IP-10, KC, MCP-1, MIP-1α, and RANTES) were above the detection limit of the assay for both mouse strains. IL-10 was detected at very low levels only in the radiation sensitive mouse strain C57BL/6 but not the radiation resistant strain C3H. The remaining cytokines (IFN-γ, IL-12(p70), IL-13, IL-15, IL-17, IL-1α, IL-1β, IL-2, IL-4, IL-5, IL-7, and TNF-α) were not detectable in the tissue lysates from either mouse strain. There was no significant difference in cytokine levels between these two strains in control animals except for G-CSF, IL-6 and IP-10, which were significantly higher level in C57BL/6 than C3H (Fig. 1).

After 12 Gy, many cytokine levels increased significantly early after radiation. There were clear differences in time-dependent changes between the two strains in 5 cytokines (G-CSF, IL-6, KC, MCP1, and IP-10) with detectable elevations (Fig. 1). All of these cytokines peaked at higher levels in C57BL/6 mice. The most striking differences occurred in levels of IL-6 which were increased by approximately 8 fold in the C57BL/6 mice but were only slightly elevated at 6 hours post radiation in the resistant C3H mice. In most cases, cytokine levels peaked 3–6 hours earlier in C57BL/6 mice.

### Cytokine levels in bronchial lavage (BAL) fluid

**Figure 2:**
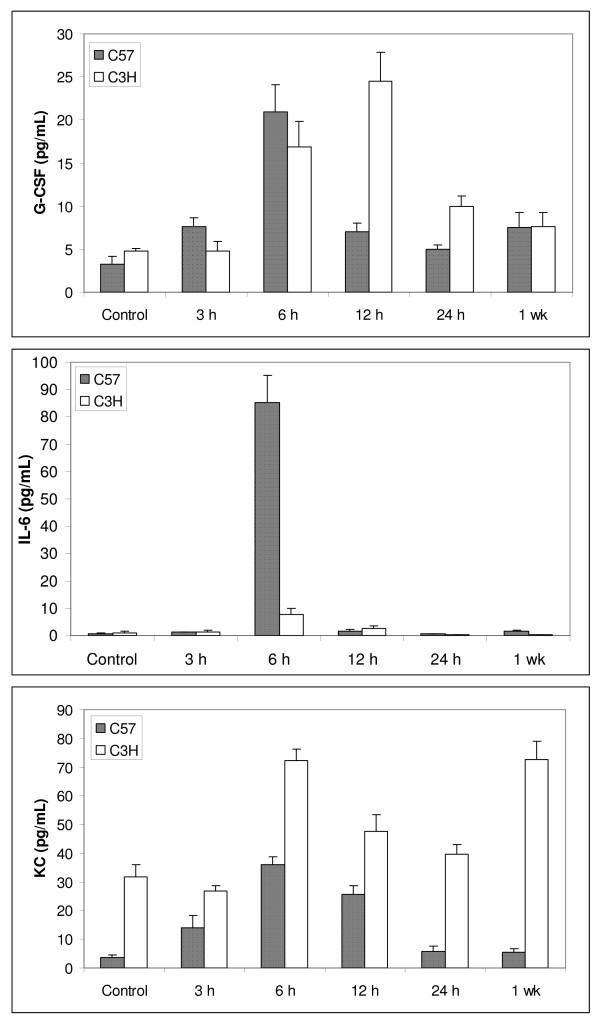
**BAL cytokine levels in C57BL/6 and C3H mice**. Mice were treated as described in Figure 1. Only three cytokines were detectable in the BAL fluid: G-CSF, IL-6, and KC. Data are expressed as the mean ± SEM of duplicate determinations from three different mice for each time point of each strain.

Only three cytokines, G-CSF, IL-6 and KC, were detectable in the BAL fluid (Fig. 2). As in lung tissue, there was no significant difference in the levels of G-CSF and IL-6 in control C57BL/6 and C3H mice, and there were radiation-induced peaks for both cytokines in both strains. The peak levels were similar for G-CSF in both strains, but the peak occurred at 6 hrs in C57BL/6 mice and at 12 hrs in C3H mice. IL-6 increased from barely detectable (<3.2 pg/ml) to approximately 90 pg/ml in the C57BL6J while the increase was minimal in the C3H. Interestingly, KC levels were significantly higher in C3H mice than in C57BL/6 mice throughout the study time course; though radiation-induced elevation was also seen in both strains.

### Cytokine levels in serum

**Figure 3:**
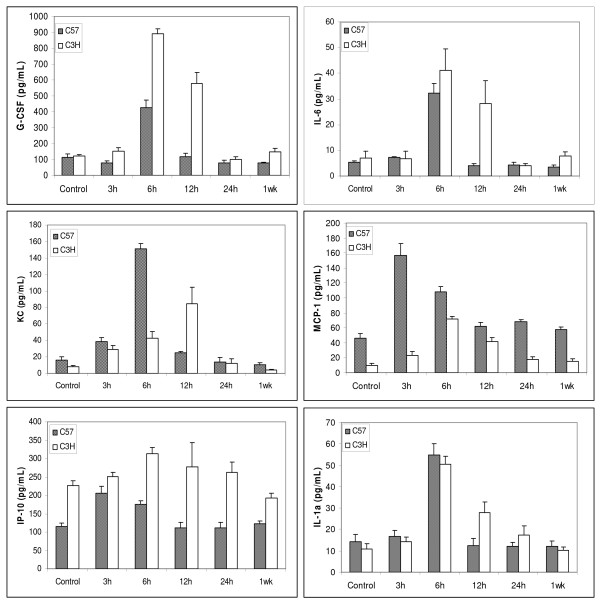
**Mouse serum cytokine levels after a single 12 Gy dose of thoracic irradiation**. Data are expressed as the mean ± SEM of duplicate determinations from three different mice for each time point of each strain.

Twelve out of 22 cytokines were above the limit of detection in the serum from both strains of mice. The detectable cytokines were G-CSF, GM-CSF, IP-10, KC, IL-6, MCP-1, IL-1α, IL-17, IL-15, IL-13, MIP-1α, and IL-12(p70). Fig. 3 shows the dynamics of cytokines with detectable changes after radiation. In control mice, G-CSF and IL-6 levels were not significantly different between these two strains. The levels of KC and MCP-1 were significantly higher, and IP-10 was lower in C57BL/6 than C3H. After radiation, the responses of the two strains were remarkably different for most of the measurable cytokines. Among the cytokines with detectable changes, the majority of them peaked 3–6 hrs earlier in C57BL/6 mice than in C3H mice. There were also significant differences in the maximum extent of elevations. MCP-1 and KC levels peaked at greater levels in C57BL/6 mice. The radiation-induced elevations were slightly greater and lasted longer for IL-6 and G-CSF in C3H mice. Radiation induced a similar level and pattern of changes in IL-1α in the two mouse strains. Thus, there were some differences in serum cytokine levels prior to radiation, and there were more significant differences in time dependent responses after radiation between these two strains.

### Relationships among cytokine levels in lung tissue, BAL fluid, and serum

There were remarkable similarities among lung tissue, BAL fluid and serum in their changing patterns of cytokine levels after radiation. A majority of the changes were characterized by a peak of elevation. The peak times of these cytokines are listed for all three types of specimen (Table 1). Of note, in C57 mice, KC peaked about 3 hours earlier in lung tissue than serum and BAL fluid. In C3H mice, G-CSF peaked about 6 hrs earlier in serum than in tissue and BAL fluid. MCP-1 and IP-10 peaked 3 hours earlier than all other detectable cytokines in both serum and tissue in both C57 mice and C3H mice.

**Table 1:** Cytokine peak time following a single dose 12 Gy whole lung irradiation for C57BL/6 and C3H mouse strains.

	C57 (hr)	C3H (hr)	Note
***Tissue***			
G-CSF	6	12	
IL-6	6	6*	*Or between 6 and12
KC	6^	6	^6 or less
MCP-1	3	6*	*Or between 6 and12
IP-10	3	18*	*Or between 12 and 24
***Serum***			
G-CSF	6	6*	*Or between 6 and 12, C3H higher peak
IL-6	6	6*	*Or between 6 and 12, C3H Higher peak
KC	6	12	
MCP-1	3	6*	*Or between 6 and 12
IP-10	3	6*	*Or between 6 and 12. Higher in C3H all time points
IL-1α	6	6*	*Or between 6 and 12
***BAL***			
G-CSF	6	12	C3H with higher peak
IL-6	6	6*	*Or between 6 and 12
KC	6	6#	# Higher in C3H all time points, peak at 6 hr to 1 wk

**Figure 4:**
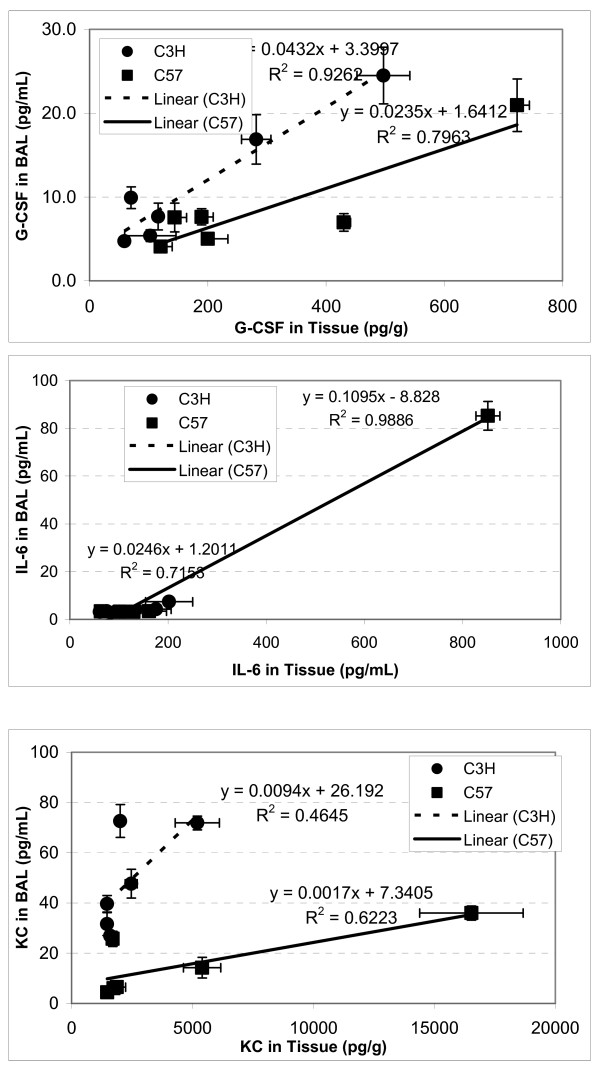
**Correlation s of cytokine levels between tissue and bronchial lavage (BAL)**. C57BL/6 (n = 18) and C3H (n = 18) mice. Error bars denote the standard errors (n = 3).

**Figure 5:**
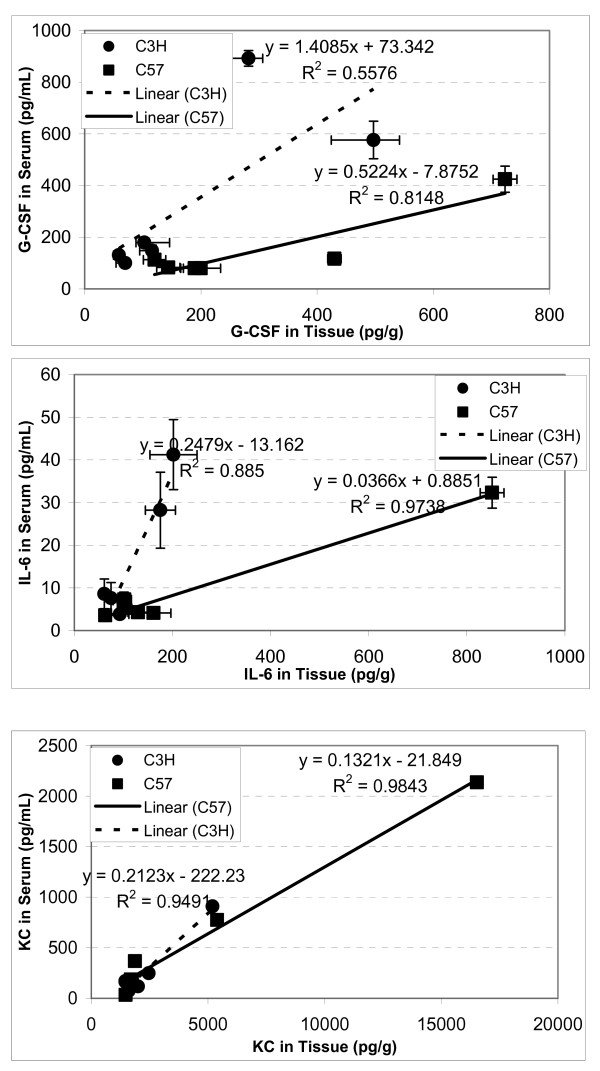
**Correlation s of cytokine levels between tissue and serum**. C57BL/6 (n = 18) and C3H (n = 18) mice. Error bars denote the standard errors (n = 3).

Among the three cytokines detectable in all three specimens, there were significant correlations of absolute levels between BAL fluid and tissue (Fig. 4), and serum and tissue (Fig. 5), though there were differences in the peak times, which caused differential changes in G-CSF and IL-6 in lung tissue/serum between C57BL/6 and C3H mice. The best correlations between serum and lung tissue levels were seen for KC, which had similar peak time in the two compartments.

## Discussion

Using a multiplex screen for 22 cytokines/chemokines at various time-points, we demonstrated significant differences after thoracic radiation in both the extent of elevation and temporal patterns in G-CSF, IL-6, and KC levels in the lung tissue, BAL fluid, and serum between two mouse strains with different sensitivity to radiation lung fibrosis. Our study is unique with respect to its measurement of early changes in multiple cytokines as well as the comparison of cytokines from primary lung tissue to BAL fluid, and serum.

The cytokines which we found to be differentially expressed in lung tissue are known to be important in initiation and maintenance of inflammatory processes. While it is impossible to discuss all the cytokines, Table 2 summarizes the specific function of each one tested here, and whether there is prior evidence of an effect of RT on its expression. For example, G-CSF increases neutrophil migration to the lung after irradiation and stimulates neutrophils to produce reactive oxygen species (ROS) and proteases, thus increasing the risk of toxicity of neutrophil products for endothelial and even epithelial cells previously injured [23,24]. G-CSF has also been reported to induce an increased synthesis of insulin-like growth factor-1 molecules by cells recruited in the lung, with possible enhancement of the fibrogenic mechanisms [25]. In our study, G-CSF peaked significantly higher in lung tissue of C57 mice, but higher in serum in C3H mice. G-CSF local levels in the lung may contribute to the radiation-induced lung damage in the C57BL/6 mouse. It is possible that G-CSF produced by local lung tissue following irradiation accumulates in the lung in the radiation sensitive mouse while local G-CSF in the lung is removed to circulating blood, which reduces the toxic effects on the lung locally in the C3H mouse. G-CSF may be an important mediator for the pathogenesis of radiation pneumonitis [26] and deserves further study in this context.

**Table 2:** Biological functions of the studied cytokines and some evidence on their expression related to radiation lung treatment.

Cytokine	Function	Prior evidence related to RT
G-CSF	Induces the survival, proliferation, and differentiation of neutrophilic granulocyte precursor cells and functionally activates mature blood neutrophils	Pulmonary toxicity^26^

GM-CSF	Stimulates the production of neutrophilic granulocytes, macrophages, and mixed granulocyte-macrophage colonies from bone marrow cells and stimulates the formation of eosinophil colonies from fetal liver progenitor cells	Elevation induced by radiation^24^

IFN-γ	Coordinates a diverse array of cellular programs through transcriptional regulation of immunologically relevant genes, antiviral and antineoplastic activity	N/A

IL-1α	Plays a role in various immune responses, inflammatory processes, and hematopoiesis.	Potential marker^4,5^; causes radiation lung toxicity^6.16,28^

IL-1β	Plays a role in immune defense against infection; induces fever, controls lymphocytes, increases the number of bone marrow cells and causes degeneration of bone joints	Uncertain correlation with RT toxicity^6^

IL-2	Causes activation and differentiation of other T lymphocytes independently of antigen	N/A

IL-4	Promotes antibody production by causing proliferation and differentiation of B-cells	N/A

IL-5	Promotes eosinophil differentiation and activation in haematopoiesis and triggering activated B-cells for a terminal differentiation into Ig-secreting cells	N/A

IL-6	Stimulates the growth and differentiation of B-cells and T-cells	Potential marker^4,5,29,30^Cause radiation lung toxicity^28^

IL-7	Promotes growth of B-cell precursors and activation of mature T-cell	N/A

IL-9	Stimulates the proliferation of erythroid precursor cells	N/A

IL-10	Co-regulates mast cell growth; inhibits synthesis of pro-inflammatory cytokines; suppresses the antigen presentation capacity of antigen presenting cells; stimulatory towards certain T cells, mast cells and B cells	Potential marker for lung toxicity^27^

IL-12p70	Involved in the differentiation of naive T cells into Th1 cells, which is important in resistance against pathogens	N/A

IL-13	Plays a role in regulating inflammatory and immune responses and has anti-inflammatory activity	Maybe related to RT lung damage, no evidence yet

IL-15	Stimulates the proliferation of T-lymphocytes; induces B-lymphocyte proliferation and differentiation.	N/A

IL-17	Induces and mediates pro-inflammatory responses; induces the production of many other cytokines, chemokines and prostaglandins from many cell types	Maybe related to RT lung damage, no evidence yet

IP-10	Selectively chemoattracts Th1 lymphocytes and monocytes and inhibits cytokine stimulated hematopoietic progenitor cell proliferation	Fibrosis related^14,32,18^

KC	Activates neutrophils and attracts neutrophils and T-lymphocytes	Fibrosis related^28^, possible marker^31^

MCP-1	Causes cellular activation of specific functions related to host defense	No correlation to RT^4^, fibrosis related^14,18^

MIP-1α	Attracts macrophages and monocytes; stimulates macrophages, and may play a role in regulating haematopoiesis	No significant correlation^18^

RANTES	Attract eosinophils, monocytes, and lymphocytes	Fibrosis sensitivity related^14,18^

TNF-α	Regulates immune cells; causes apoptotic cell death, cellular proliferation, differentiation, inflammation, tumorigenesis, and viral replication; induces necrosis (death) of tumor cells and possesses a wide range of proinflammatory actions	Causes radiation-induced lung toxicity^22,28,37^

MIP-1β	Attracts macrophages and monocytes; stimulates macrophages and acute lung inflammation	RT lung injury^38^

Likewise, IL-6 was up-regulated and peaked at 6 hrs after radiation in lung tissue, BAL fluid and blood in C57BL/6 mice (Fig. 1, Table 1), which is somewhat consistent with previous reports [4,5,22-27]. IL-6, a major mediator of the acute-phase inflammatory response, can be synthesized by a variety of cells in the lung parenchyma such as fibroblasts and alveolar macrophages and has been found to be upregulated within hours following ionizing radiation [28]. High levels of IL-6 in the C57BL/6 mouse lung (8-fold increase compared with 2.8-fold in C3H mouse 6 hrs post-irradiation) may exacerbate the inflammatory response in the lung (overreacting), which ultimately causes IL-6 leakage to bronchoalveolae and further lung damage. Thus, IL-6 removal from local lung tissue to circulating blood might help reduce the IL-6 overreacting inflammatory response and play a protective role in the C3H mouse lung. Additionally, the tight correlation (R^2 ^= 0.97) between tissue and serum levels suggests that blood IL-6 could be a good predictor for radiation pneumonitis [4,29,30].

KC is a neutrophil and monocyte chemoattractant and the murine functional homolog of human IL-8, and blood IL-8 level has been reported to have predictive value for symptomatic radiation-induced lung injury in patients receiving thoracic radiation [31]. Our study demonstrated significant elevations in KC level after radiation, and we found a significant correlation between blood and tissue levels. During acute lung inflammation, KC produced primarily by pulmonary fibroblasts acts in chemotaxis and activation of neutrophils. Also, IL-8 has been implicated as a significant angiogenic factor in idiopathic pulmonary fibrosis [32]. Our data further confirm that KC is most probably produced locally from the lung, as it peaked approximately 3–6 hours earlier in tissue than in blood of both C57BL/6 and C3H mice. The higher level of KC working together with other inflammatory cytokines such as IL-6 and G-CSF may attract more inflammatory cells such as neutrophils, monocytes, macrophages to the injured local lung in the C57BL/6 mouse, which ultimately causes serious damage to the lung and leads to chronic fibrosis [14].

While our study focused on cytokine protein levels, previous studies have documented radiation-induced changes in cytokine mRNA expression in these two mouse strains and have shown a biphasic expression in the lung: an initial transitory cytokine response and a second more persistent cytokine mRNA elevation [33]. In other work, Chiang et al. reported that both BAL and whole lung tissue showed biphasic cytokine mRNA responses with striking temporal differences between the two compartments and changes in the lung tissue correlating better than BAL with the onset of fibrosis in the C57BL/6 mouse strain during the latent period [34]. Also, Hong et al. reported early differences between these two mouse strains [20] in mRNA of IL-6 and TNFα following lung irradiation. The lack of agreement between our and Hong et al's data might be due to the poor correlation between mRNA levels derived from gene expression and protein expression levels, which can vary up to 20-fold [35,36].

The remarkable dynamic changes in cytokine levels suggest that the timing of changes in cytokine levels may be particularly important. In most cases, in lung tissue and BAL fluid, cytokine levels increased earlier in the more sensitive strain than in the more resistant strain. As these changes were relatively transient, the meaning of the earlier increase in C57BL/6 is unknown. However, these data do suggest that the time after radiation when measurements are taken should be considered in the development of a predictive assay. Furthermore, both the correlation in levels between tissue and more easily accessible sites such as BAL or serum and the predictive value must be considered. For example, both G-CSF and IL-6 had greater and earlier peaks in lung tissue and BAL fluid in the more sensitive C57/BL6 mice, but in serum the peak levels were greater for these molecules in the more resistant C3H mice. On the other hand, tissue KC and serum KC are more positively correlated than are tissue and BAL KC. Further study is needed to further investigate the potential mechanisms and the values of these molecules in predicting long term toxicity.

This study has some limitations. Although it provides a high throughput and reproducible measurements, this multiplex cytokine assessment is not optimized for measurement of all cytokines. Of note, only 9, 3, and 12 cytokines were measurable in lung tissue, BAL fluid and serum, respectively. The inability to detect other cytokines may be due to the detection limits of the assay in addition to un-optimized assay conditions. For instance, TNF-α may be involved in the generation of radiation-induced lung damage [37] but was at the borderline for its detection. Also, we chose serum as we were initially interested in TNF-α level; however, the use of serum instead of plasma may have resulted in measurements of cytokines that were released from platelets during coagulation thus making the results more difficult to interpret. In addition, TGF-β1, known to play a major role in the lung's response to radiation by other studies, was not measured in our study due to the limited blood sample and the absence of plasma samples.

In summary, this study demonstrates that thoracic radiation induced significant strain-dependent early expressions of G-CSF, IL-6, and KC in the lung tissue, BAL fluid, and serum in C3H/HeN and C57/BL6 mice. Correlations between levels in tissue and blood suggest the possibility of using blood as a surrogate marker to estimate or predict tissue changes and thus late radiation toxicity. Further study is needed to elucidate the underlying mechanism of such differences and determine which of the earlier changes may be predictive of pneumonitis or late fibrosis.

## Competing interests

The authors warrant that there is no conflict of interests, including conflicts of a financial nature involved with any pharmaceutical company.

## Authors' contributions

XA designed and performed experiments, analyzed the results and wrote the manuscript; LZ performed the animal experiments; MAD helped with experiments, data interpretation and manuscript preparation; DML provided materials and helped manuscript preparation; TSL provided funding, oversaw all steps of experiments, helped with data analysis and interpretation, and involved in the manuscript preparation; FMK as senior author involved in the experimental design, data interpretation, manuscript preparation, and approved the final document.
